# Life course models: improving interpretation by consideration of total effects

**DOI:** 10.1093/ije/dyw329

**Published:** 2016-12-12

**Authors:** Michael J Green, Frank Popham

**Affiliations:** * MRC/CSO Social & Public Health Sciences Unit, University of Glasgow, Glasgow, UK

**Keywords:** Life course, mediation, causality

## Abstract

Life course epidemiology has used models of accumulation and critical or sensitive periods to examine the importance of exposure timing in disease aetiology. These models are usually used to describe the direct effects of exposures over the life course. In comparison with consideration of direct effects only, we show how consideration of total effects improves interpretation of these models, giving clearer notions of when it will be most effective to intervene. We show how life course variation in the total effects depends on the magnitude of the direct effects and the stability of the exposure. We discuss interpretation in terms of total, direct and indirect effects and highlight the causal assumptions required for conclusions as to the most effective timing of interventions.

**Key Messages**

- Life course models that consider the total effects of exposure improve interpretations regarding the most effective timing of interventions.
- The magnitude of direct effects and the stability of exposure are both important determinants of how the total effects of exposure vary over the life course.
- Interpretations from life course models regarding effective timing of interventions require a range of causal assumptions.

## Introduction

In this article we explain how interpretation of life course models can change when total effects are considered in addition to direct effects, giving a clearer idea of when intervention may be most effective and of the pathways an intervention needs to act on. The article covers principles of how the direct, indirect and total effects of a repeated exposure are related. It concludes discussing how the total, direct and indirect effects can inform the timing and evaluation of interventions, and the assumptions regarding confounding that are required. This article focuses on concepts and is intended as supplementary to more mathematical/technical treatments of how total, direct and indirect effects should be estimated.^1–4^

**Figure 1.**
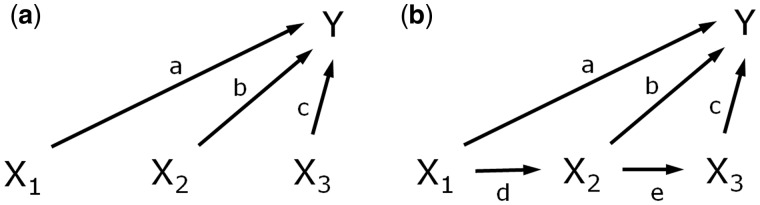
Health and risk exposures over time.

A starting point for life course epidemiology is the models of accumulation and critical or sensitive periods.^5^Figure 1a describes these models, showing an exposure X having an influence on a health outcome Y. X is measured at three time points, at different life stages. The paths a, b and c refer to the effects of X at each time point on Y, that is, the expected change in Y caused by a unit change in X at that time point. Accumulation models suggest that timing is irrelevant (i.e. a = b = c): an exposure has an equal effect whenever it is experienced and thus it is the duration, rather than the timing of exposure, that is important. Sensitive period models suggest an exposure has a heightened effect at one time compared with other times (e.g. a > b and b = c). This can be generalized to allow for variation in the effect of the exposure over time (e.g. a > b > c). Critical period models posit that timing is vital: an exposure only has an effect if experienced within a particular time window (e.g. a > 0 but b = c = 0, i.e. a special case of the sensitive period model).

Accumulation and critical/sensitive period models describe variation in the magnitude of effects over the life course, typically referring to the direct effects of exposure. Further elaboration of these models has included links between exposures over time. This is illustrated by paths d and e in Figure 1b, representing the effects on X at a later time point from X at earlier times. This has allowed for further ‘pathways’ models such as the ‘chain of risk trigger model’, where a = b = 0, and c, d and e are all > 0, or the ‘chain of risk additive model’ where all paths have effects > 0.^5^^,^^6^ Such pathways models are useful for any exposure that tracks over time [examples could include smoking, body mass index (BMI), mental health, socioeconomic disadvantage].

Acknowledging paths d and e means life course models can be placed in a mediation framework,^7^ allowing for a distinction between total, direct and indirect effects. The total effect of exposure X_1_ on Y, for example, can be decomposed into a direct effect of X_1_, and indirect effects via X_2_ and X_3_.^1^^,^^2^^,^^4^^,^^8^ If the arrows in Figure 1 represent regression coefficients, with a-c coming from the same model for Y, the direct effect of X_1_ = a, whereas indirect effects are calculated by multiplying the relevant coefficients (i.e. d*b for the indirect effect of X_2_ and d*e*c for X_3_). The sum of these direct and indirect effects then gives the total effect of X_1_ on Y. This is known as the ‘product of coefficients’ method and holds when all variables are continuous and are related in a linear fashion without interactions and none of the associations are confounded.^2^^,^^9^^,^^10^ Coefficients from such models are often standardized, but these principles apply regardless of metric.^8^ These may be restrictive conditions (and we return to these later), but this simple case is useful conceptually. Seen this way, it is recognizable that the terms of accumulation or critical/sensitive periods might alternatively describe variations in the magnitude of the total effects of exposure over the life course, rather than variation in the direct effects only.

## Interdependence of total and direct effects

Knowing the direct effects of an exposure allows some inference regarding life course variation in the total effects, as shown in Table 1. The implied life course variation in total effects is sometimes different from life course variation in direct effects. Assume that paths d and e in Figure 1b have coefficients somewhere between 0 and 1, representing some but not total stability in the exposure over time (see footnote to Table 1). If the direct effects follow an early critical period model where a > 0 but b = c = 0, then the total effects will also follow an early critical period model. Since b and c are both 0, there are no indirect effects and the total effect of X_1_ = a. X_2_ and X_3_ will have total effects equal to 0 because they can only influence Y via paths involving b or c. However, if the direct effects follow a late critical period model (c > 0 but a = b = 0), then the total effects follow a sensitive period model where the later exposure X_3_ has the greatest total effect, but X_1_ and X_2_ also have total effects, albeit weaker than for X_3_ because they are indirect.

**Table 1.** Life course models for total effects implied by different models of direct effects where exposures track over time

Life course model for direct effects	Total effect			Implied life course model for total effects^a^
	X_1_ = a + (d*b) + (d*e*c)	X_2_ = b + (e*c)	X_3_ = c	
Early critical period (a > 0 and b = c = 0)	a	0	0	Early critical period
Late critical period (c > 0 and a = b = 0)	d*e*c	e*c	c	Late sensitive period d*e*c < e*c < c
Accumulation (a = b = c)	a + (d*a) + (d*e*a)	a + (e*a)	a	Early sensitive period a + (d*a) + (d*e*a) > a + (e*a) > a
Early sensitive period (a > b > c)	a + (d*b) + (d*e*c)	b + (e*c)	c	Early sensitive period a + (d*b) + (d*e*c) > b + (e*c) > c
Late sensitive period (a < b < c)	a + (d*b) + (d*e*c)	b + (e*c)	c	Depends on the relative magnitude of paths a-e

^a^Assuming 0 < d < 1 and 0 < e < 1; outside this simplifying assumption the conclusions in this column may not always hold. Specifically, with coefficients > 1 a late critical period model for the direct effects implies an early rather than a late sensitive period model for the total effects, whereas other conclusions hold. With coefficients < 0, which would be unusual for a repeated exposure, the pattern of total effects will depend on the relative magnitude of the paths a-e.

If the direct effects follow an accumulation model where a = b = c, then the total effects will follow a sensitive period model where earlier exposures have progressively stronger total effects. This is because earlier exposures combine indirect effects through later exposure with the direct effects of exposure. Similarly, earlier exposures will also have stronger total effects than later exposures where the direct effects follow an early sensitive period model (i.e. a > b > c), though the relative difference between the total effects of earlier and later exposures will be more dramatic. However, if the direct effects follow a late sensitive period model (a < b < c), then the relative magnitude of the total effects depends on the relative magnitude of the direct effects (a-c) and the stability of the exposure (d-e). Where direct effects follow early sensitive/critical period or late critical period models, inferences from total effects regarding when to intervene will be concordant with those from direct effects. However, for accumulation and some late sensitive period models of direct effects, inferences from total effects regarding when to intervene will be discordant with those from direct effects. Further, Table 1 shows that where direct effects follow accumulation or early sensitive/critical period models, total effects will be stronger for earlier than for later exposures. Only for late critical/sensitive period models of the direct effects can total effects be stronger for later than for earlier exposures.

For many models shown in Table 1, the degree to which the total effects emphasize earlier or later exposures will depend on the magnitude of the direct effects (a-c) and the stability of the exposure (paths d-e). Table 2 uses some arbitrary effects sizes (labelled ‘low’, ‘mid’ and ‘high’ relative to each other) to demonstrate what happens when the magnitude of the direct effects changes (keeping the stability of the exposures constant). The last row of Table 2 compares the total effect of X_1_ to that of X_3_. The total effect of X_1_ is always larger than that of X_3_ for accumulation and early sensitive or critical period models, but as the magnitude of the direct effects ranges from low to high, the difference between the total effects of X_1_ and of X_3_ increases. In contrast, for the late sensitive period model where later exposures have larger effects, the relative importance of X_1_ and X_3_ can reverse as the magnitude of the direct effects ranges from low to high. In the provided example, where direct effects are small X_3_ has a larger total effect than X_1_, with medium direct effects X_1_ and X_3_ are almost equivalent, and where direct effects are large X_1_ has a larger total effect than X_3_. This is not inevitable but additionally depends on the relative magnitude of the direct effects. Where later exposures have very strong direct effects relative to earlier ones (e.g. a late critical period model), stronger direct effects will result in the total effect of X_3_ being stronger relative to X_1_.

**Table 2.** Comparison of total effects when the magnitude of direct effects varies

Life course model for direct effects	Accumulation (a = b = c)			Early sensitive period (a > b > c)			Early critical period (a > 0; b = c = 0)			Late sensitive period (a < b < c)			Late critical period (a = b = 0; c > 0)		
Magnitude of direct effects	Low	Mid	High	Low	Mid	High	Low	Mid	High	Low	Mid	High	Low	Mid	High
Effect sizes:															
a	0.3	0.5	0.7	0.5	0.7	0.9	0.5	0.7	0.9	0.1	0.3	0.5	0.0	0.0	0.0
b	0.3	0.5	0.7	0.3	0.5	0.7	0.0	0.0	0.0	0.3	0.5	0.7	0.0	0.0	0.0
c	0.3	0.5	0.7	0.1	0.3	0.5	0.0	0.0	0.0	0.5	0.7	0.9	0.5	0.7	0.9
d	0.5	0.5	0.5	0.5	0.5	0.5	0.5	0.5	0.5	0.5	0.5	0.5	0.5	0.5	0.5
e	0.5	0.5	0.5	0.5	0.5	0.5	0.5	0.5	0.5	0.5	0.5	0.5	0.5	0.5	0.5
Total from X_1_	0.53	0.88	1.23	0.68	1.03	1.38	0.5	0.7	0.9	0.38	0.73	1.08	0.13	0.18	0.23
Total from X_2_	0.45	0.75	1.05	0.35	0.65	0.95	0.0	0.0	0.0	0.55	0.85	1.15	0.25	0.35	0.45
Total from X_3_	0.30	0.50	0.70	0.10	0.30	0.50	0.0	0.0	0.0	0.50	0.70	0.90	0.50	0.70	0.90
Difference (X_1_-X_3_)	0.23	0.38	0.53	0.58	0.73	0.88	0.5	0.7	0.9	−0.13	0.03	0.18	−0.38	−0.53	−0.68

Table 3 demonstrates what happens when the stability of the exposure changes which, with direct effects kept constant, can only impact on the total effects via the indirect effects. Where the direct effects follow an accumulation or early sensitive period model, the total effect of X_1_ is larger than that of X_3_ and this difference widens when exposures are more stable over time. For early critical period models, there are no indirect effects so changing the stability makes no difference. The late sensitive period example shows that the stability of the exposure can reverse the direction of the difference between the total effects of X_1_ and X_3_. In the late critical period example, the relative importance of X_3_ still diminishes as stability increases, but the direction of the difference does not necessarily reverse.

**Table 3.** Comparison of total effects when the stability of exposures varies

Life course model for direct effects	Accumulation (a = b = c)			Early sensitive period (a > b > c)			Early critical period (a > 0; b = c = 0)			Late sensitive period (a < b < c)			Late critical period (a = b = 0; c > 0)		
Stability of exposures	Low	Mid	High	Low	Mid	High	Low	Mid	High	Low	Mid	High	Low	Mid	High
Effect sizes:															
a	0.5	0.5	0.5	0.7	0.7	0.7	0.7	0.7	0.7	0.3	0.3	0.3	0.0	0.0	0.0
b	0.5	0.5	0.5	0.5	0.5	0.5	0.0	0.0	0.0	0.5	0.5	0.5	0.0	0.0	0.0
c	0.5	0.5	0.5	0.3	0.3	0.3	0.0	0.0	0.0	0.7	0.7	0.7	0.7	0.7	0.7
d	0.3	0.5	0.7	0.3	0.5	0.7	0.3	0.5	0.7	0.3	0.5	0.7	0.3	0.5	0.7
e	0.3	0.5	0.7	0.3	0.5	0.7	0.3	0.5	0.7	0.3	0.5	0.7	0.3	0.5	0.7
Total from X_1_	0.70	0.88	1.10	0.88	1.03	1.20	0.7	0.7	0.7	0.51	0.73	0.99	0.06	0.18	0.34
Total from X_2_	0.65	0.75	0.85	0.59	0.65	0.71	0.0	0.0	0.0	0.71	0.85	0.99	0.21	0.35	0.49
Total from X_3_	0.50	0.50	0.50	0.30	0.30	0.30	0.0	0.0	0.0	0.70	0.70	0.70	0.70	0.70	0.70
Difference (X_1_-X_3_)	0.20	0.38	0.60	0.58	0.73	0.90	0.7	0.7	0.7	−0.19	0.03	0.29	−0.64	−0.53	−0.38

## Inference from total, direct and indirect effects

Whether a particular period is sensitive in terms of total effects only or in terms of total and direct effects has implications for theoretical explanations of how the exposure acts on the outcome. For example, adolescence is receiving increasing attention as a potential sensitive period.^11^^,^^12^. Say X_1_ represents smoking during adolescence, X_2_ and X_3_ represent smoking later in life and Y represents breast cancer. A Canadian study found that initiating smoking during puberty was associated with increased risk of breast cancer in women after adjusting for life course smoking duration.^13^ This implies that adolescence (or puberty at least) is a sensitive period for the direct effects of smoking, requiring a theoretical explanation for the heightened direct effect in adolescence (e.g .enhanced carcinogenic effects of smoking on rapidly dividing tissue during pubertal development^513^). Had there been no effect of pubertal initiation after adjusting for smoking duration, this would imply an accumulation model for the direct effects: there would be no need to postulate theories for sensitive direct effects.

Nevertheless, in the smoking example above, if initiation of smoking in adolescence leads to a greater risk of smoking in adulthood, adolescence should be interpreted as a sensitive period in terms of total effects regardless of sensitivity in direct effects. It will still be a key point for intervention, and this message may be missed by focusing only on the direct effects. Similarly, examples abound where socioeconomic disadvantages are associated cumulatively with greater risks for mortality or poor health.^14^^,^^15–18^ If later disadvantages are more likely because of earlier ones and the direct effects of socioeconomic disadvantages are more or less equal, then earlier disadvantages will have stronger total effects than later ones, and might be considered sensitive periods for intervention.

With an interest in when it will be most efficient to intervene, the total effects are relevant as they indicate when the effects of intervening could be maximized. If adolescence is a sensitive period, then this may be the best time to intervene, regardless of whether it is sensitive in terms of total effects only or in terms of both total and direct effects. Compared with looking only at the direct effects, total effects will tend to direct toward intervention earlier in life, as for most combinations of effect sizes the total effects will be stronger for earlier than for later exposures. Considering total effects also recognizes how exposure stability influences conclusions regarding efficient intervention timing: where early exposures are strong determinants of later exposure, total effects will be stronger earlier in life (excepting critical period patterns of direct effects). However, the relative strength of the direct and indirect contributions to the total effects highlights the relative importance of the paths the intervention should act on. Taking the smoking example from above, if most of the effect of smoking in adolescence is indirect through increased duration of smoking in adulthood, then a key criterion for evaluating interventions on adolescent smoking will be the extent to which they reduce the risk of prolonged smoking in adulthood. Conversely, if most of the effect were direct via a sensitive direct effect, interventions could focus simply on adolescent smoking and ignore adult smoking.

## Confounding

**Figure 2.**
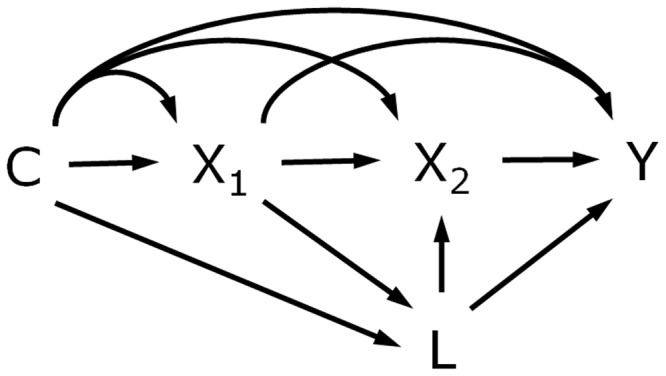
Confounding for effects of repeated exposures.

As noted, a model such as that in Figure 1b is essentially a mediation analysis (e.g. with X_2_ and X_3_ as mediators of X_1_). Whatever analysis method is used, confounding should be considered.^2^^,^^19–21^ Consider Figure 2, which is a directed acyclic graph (DAG) of confounding in mediation^2^ replacing the mediator with a second measurement of the exposure. DAGs can be useful tools for representing the assumed causal structure underlying the data and identifying possible confounders *a priori* so that they can be measured and adjusted for.^11^^,^^19^ Causal inference requires appropriate adjustment for relevant confounders and assumes no influence from unmeasured confounders.^22^ Specifically, if we refer to X_1_ as the exposure and X_2_ as the mediator, four assumptions regarding confounding might be required for causal interpretation: (i) no unmeasured confounding of the exposure-outcome relationship (i.e. X_1_ to Y); (ii) no unmeasured confounding of the mediator-outcome relationship (i.e. X_2_ to Y); (iii) no unmeasured confounding of the exposure-mediator relationship (i.e. X_1_ to X_2_); and (iv) no mediator-outcome confounders that are affected by the exposure (as depicted by L in Figure 2^1^^,^^2^^,^^10^). For assumptions (i)-(iii), sensitivity analyses can be used to identify how large the effect of unmeasured confounders would have to be to negate observed findings.^1^^,^^2^^,^^10^ Total effects only require assumption (i), whereas direct and indirect effects require more, depending on the specific effect being estimated.^3^

Even if measured and adjusted for, mediator-outcome confounders that are affected by the exposure (L) can be problematic because such variables are both confounders and mediators.^22^ Adjusting can remove a part of the ‘direct’ effect of the exposure (i.e. the effect not via the mediator) and not adjusting can introduce collider-bias.^1^ Solutions are available using special techniques and/or further assumptions^1^^,^^3^^,^^23^ or alternatively, an L-type confounder might be viewed as an additional mediator with indirect effects fully decomposed into those via X_2_ alone, those via L alone and those via L and X_2_.^4^ It may not always be clear which type of confounding is present, e.g. where X_1_ and a mediator-outcome confounder are measured concurrently, but analysing the data under different assumptions (e.g. C- or L-type) can show how sensitive conclusions are to those assumptions.

Consider briefly what assumption (iii), no unmeasured confounding of the exposure-mediator relationship (i.e .X_1_ to X_2_), means within the repeated-exposure context. Effects of early on later exposures are assumed to be causal, i.e. exposure status at one point in time has a causal influence on later exposure status: a change in the earlier status will produce a change in later status. This will be especially important where strong total effects of early exposures are largely mediated indirectly through later exposures. For example, if X represents income, and early life income has strong effects largely mediated through income stability, we might wish to intervene to raise income in early life. If the association between early and later life income is causal then this may be effective, but if this association is confounded by other factors then raising early life income will not affect later life income. It will fail to activate the strong indirect paths to Y, leaving only the weaker direct effect of early life income. As another example, diet and exercise interventions can be effective in producing short-term weight loss, but losses are often not maintained^24^^,^^25^ and so their impact on cardiovascular disease outcomes may be limited. If an intervention fails to address the underlying causes of an exposure trajectory, that trajectory may not be altered over the long term and any potential for large indirect effects via reduced exposure across that trajectory will be lost.

## Estimation

Traditional mediation analysis involves estimation of a series of regression models, with the indirect effect calculated by multiplying relevant coefficients together, or alternatively by examining the difference in the coefficient of the exposure before and after the mediator is added to the model.^1^^,^^2^^,^^7^^,^^10^ Supported by consideration of confounding as described above, these approaches will be valid (and mathematically equivalent) where variables are related in linear fashion without interactions.^2^^,^^3^^,^^10^ If the outcome or mediator is binary then similar methods employing logistic regressions may also be valid, but if a binary outcome is not rare neither method will be accurate as odds ratios are not collapsible.^2^^,^^26^ A further traditional alternative is to estimate all associations jointly as a series of simultaneous equations (e.g. path analysis, structural equation modelling^27^), which again relies on assumptions of linearity and no interactions.^4^^,^^10^

More recent developments in the causal inference literature include more precisely decomposed definitions of direct and indirect effects, which allow for interactions between the exposure and mediator,^2–4^^,^^10^ and flexible simulation methods to overcome constraints regarding no interactions and linearity.^2^^,^^10^ As researchers consider finer decompositions of total effects that explicitly include interactive effects of exposures and mediators, we recommend that a thoughtful comparison of the decomposed and total effects will remain valuable, as we have shown it to be in the simple case here. This is particularly relevant for life course models where there are multiple measures of exposure (or multiple mediators), as this dramatically increases the complexity of effect decomposition.^4^ Further research as to how the principles introduced here would apply in more complex situations would be worthwhile.

## Conclusion

Distinguishing total, direct and indirect effects can aid understanding of life course models where exposures track over time. Inferences about when it may be most efficient to intervene should take into account total, direct and indirect effects. Applying mediation techniques to life course models with a view to causal inference requires full consideration of confounding, including confounding of the direct effects of repeated exposures [i.e. assumptions (i), (ii) and (iv) above] and confounding of exposure stability [assumption(iii)].

## Funding

This work was supported by the UK Medical Research Council [grant number MC_UU_12017/13] and the Scottish Government Chief Scientist Office [grant number SPHSU13].

**Conflict of interest:** None declared.
